# Endovenous chemical ablation and trendelenburg’s (eCAT) operation for treating great saphenous vein varicosities: a single-arm open-label interventional study

**DOI:** 10.1007/s00423-025-03964-6

**Published:** 2026-02-04

**Authors:** Walied Khereba, Al Metwaly Ragab, Ayman Amen MohyElden, Khaled Attia, Osama Moeen, Mohamed Ahmed Agena, Elsayed Hadhoud, Amr Bakr Mahmoud Elashry, Ahmed Atef, Maisa A. Abdel Wahab, Nehal Farouk, Sameh E. Elimam, Ahmed Khairy Sakr, Waleed E. Elshinawy, Shimaa M. Elhalafawy, Ahmed Ibrahim Badran, Ahmed MT Radwan, Mohamed Emad Eldin, Mohamed Yahia Zakaria, Reda Othman Abbas, Hussien Montaser, Elsayed Mohamed Abd El-Hamid, Rasha S. Farag

**Affiliations:** 1 Department of Vascular and Endovascular Surgery, Damietta Faculty of Medicine, Al-Azhar University, New Damietta, Damietta Egypt; 2 Department of Vascular and Endovascular Surgery, Assuit Faculty of Medicine, Al-Azhar University, Assuit, Egypt; 3 Department of Vascular and Endovascular Surgery, Faculty of Medicine for Girls, Al-Azhar University, Cairo, Egypt; 4 Department of Vascular and Endovascular Surgery, Faculty of Medicine, Tanta University, Al-Gharbia, Egypt; 5 Department of Vascular Access & Renal Transplant Surgery, Birmingham University Hospital, Birmingham, UK; 6 Department of Vascular and Endovascular Surgery, Faculty of Medicine, Mansoura University, Mansoura, Egypt; 7 Department of Vascular and Endovascular Surgery, Faculty of Medicine for Boys, Al-Azhar University, Cairo, Egypt; 8 Department of Radio-diagnosis, Faculty of Medicine for Boys, Al-Azhar University, Cairo, Egypt; 9 Department of Radio-diagnosis, Damietta Faculty of Medicine, Al-Azhar University, Damietta, New Damietta Egypt; 10 Department of Clinical Research, Damietta Directorate for Health Affairs, Egyptian Ministry of Health and Population, Egyptian Ministry of Health and Population, Egypt

**Keywords:** Endovenous chemical ablation, Trendelenburg operation, eCAT, Great saphenous vein, Foam sclerotherapy

## Abstract

**Background:**

Varicose veins of the lower limbs, particularly involving the great saphenous vein (GSV), are a common vascular condition often requiring intervention. Conventional surgeries entail higher morbidity and prolonged recovery. To minimize complications while ensuring efficacy, a novel hybrid technique —Endovenous Chemical Ablation and Trendelenburg’s operation (eCAT) —was developed. This study aimed to evaluate the efficacy and safety of the eCAT operation for treating GSV varicosities.

**Methods:**

A single-arm open-label interventional study was conducted on 500 patients with primary lower limb varicose veins (2014–2021). The eCAT procedure, developed by Walied Khereba at Al-Azhar University (New Damietta), combined Trendelenburg’s operation with polidocanol foam sclerotherapy under local anaesthesia. The primary outcome was GSV ablation efficacy at 1 week, 3 months, and 1 year. Secondary outcomes included postoperative complications, pain reduction, and factors influencing success.

**Results:**

GSV ablation succeeded in all cases (100%). At 1 week, 88% of veins were fully occluded and 12% partially; at 3 months, full occlusion decreased to 76%, then improved to 88% at 1 year. Postoperative complications included oedema (30%), hyperpigmentation (20%), and residual varicosities (40%), all resolved by 1 year. Median pain scores decreased from 4 (IQR: 1) preoperatively to 2 (IQR: 1) at 1 week and 1 (IQR: 0) at both 3 months and 1 year (*P* = 0.001). Age, gender, and standing occupation significantly impacted early surgical success.

**Conclusion:**

The eCAT is a safe, effective, and minimally invasive procedure for GSV varicosities treatment, achieving durable clinical outcomes with minimal complications and significant pain reduction.

## Introduction

Chronic Venous Disease (CVD) is a condition characterized by chronic venous insufficiency, often caused by blood pooling in the veins or strain on the vein walls [1, 2]. One of the primary causes of venous insufficiency is reflux in superficial veins, particularly the great saphenous vein (GSV) [3]. In most cases, this condition can be effectively treated with procedures such as endovenous chemical or laser ablation, radiofrequency ablation, sclerotherapy, or venous arterialization of varicose veins (VV) [4–6]. However, CVD can have a profound and lasting impact on an individual’s life, affecting financial, physical, mental, and emotional well-being [7].

Chronic venous insufficiency frequently leads to damage in the veins of the legs, causing them to enlarge and twist, thereby impairing their ability to transport blood back to the heart [8]. This condition, known as venous varicosity, can contribute to cardiovascular complications and increased venous pressure [9]. Factors contributing to VV development include genetic predisposition, faulty valves, weakened vein walls, and elevated vein pressure [10, 11]. While the exact cause of VV remains debatable, these factors play a significant role in their formation. Additionally, the risk of VV can be attributed to other factors, such as lifestyle and dietary habits [9]. Women are more prone to venous insufficiency due to factors such as multiple pregnancies, obesity, and prolonged standing, although the condition affects both genders [2]. Saphenous vein malfunction often underlies this issue, leading to complications ranging from cosmetic concerns to ulcerations, with a prevalence of 40% in women and 20% in men, highlighting its significant public health impact [12].

Treatment options for GSV incompetence include high ligation, vein stripping, laser ablation, radiofrequency ablation, and ultrasound-guided foam sclerotherapy [13]. Foam sclerotherapy, which enhances contact between the vein wall and the injected agent, may be less effective in larger veins with significant blood flow due to agent dispersion. For patients with GSV diameters exceeding 2 cm, triple saphenectomy—a combination of saphenofemoral disconnection, GSV stripping, and stab avulsion—may be the preferred treatment approach [14]. The Endovenous Chemical Ablation and Trendelenburg’s (eCAT) operation is a minimally invasive technique designed to treat GSV varicosities. It was developed by Walied Khereba and his team in April 2014 at Al-Azhar University Hospital (New Damietta). This procedure combines the principles of Trendelenburg’s operation, which involves the flush disconnection of the Saphenofemoral Junction (SFJ), with chemical ablation using polidocanol foam. In this study, we aimed to evaluate the efficacy and safety of eCAT operation in treating GSV varicosities by assessing the success rate of GSV ablation, post-operative complications, and pain reduction, while also analyzing the impact of patient demographics, job-related factors, and other variables on treatment outcomes.

## Methods

### Study design and setting

This single-arm, single-center, interventional study was conducted over a period of seven years, from April 2014 to April 2021, at Al-Azhar University Hospital (New Damietta). The study was performed in accordance with the Declaration of Helsinki ethical principles and received approval from the Ethics Committee of the Faculty of Medicine, Al-Azhar University, New Damietta (DFM-IRB 00012367-22-06-004). Informed consent was obtained from all patients before their participation. The study protocol has been registered at ClinicalTrials.gov under the number (NCT05655416).

### Study population

Adult patients with primary lower limb VV were included in the study. Exclusion criteria included pregnant women, individuals with secondary or recurrent VV, lymphedema, acute superficial thrombophlebitis, arteriovenous fistula (congenital or acquired), congenital anomalies of the venous system, skin infections, lower limb ischemia, and a history of allergic reactions or known hypersensitivity to polidocanol or similar agents.

### Data collection

Comprehensive medical histories were recorded for all patients, followed by thorough clinical assessments of symptoms and lesions. No routine preoperative screening for right-to-left cardiac shunts, such as patent foramen ovale (PFO), was performed. Laboratory and radiological investigations, particularly Duplex ultrasound of the venous drainage of the lower limbs, were performed to evaluate the GSV, small saphenous vein, and extra-truncal varicosities. Vital signs were documented prior to the procedure.

### Procedure of eCAT

**Fig. 1:**
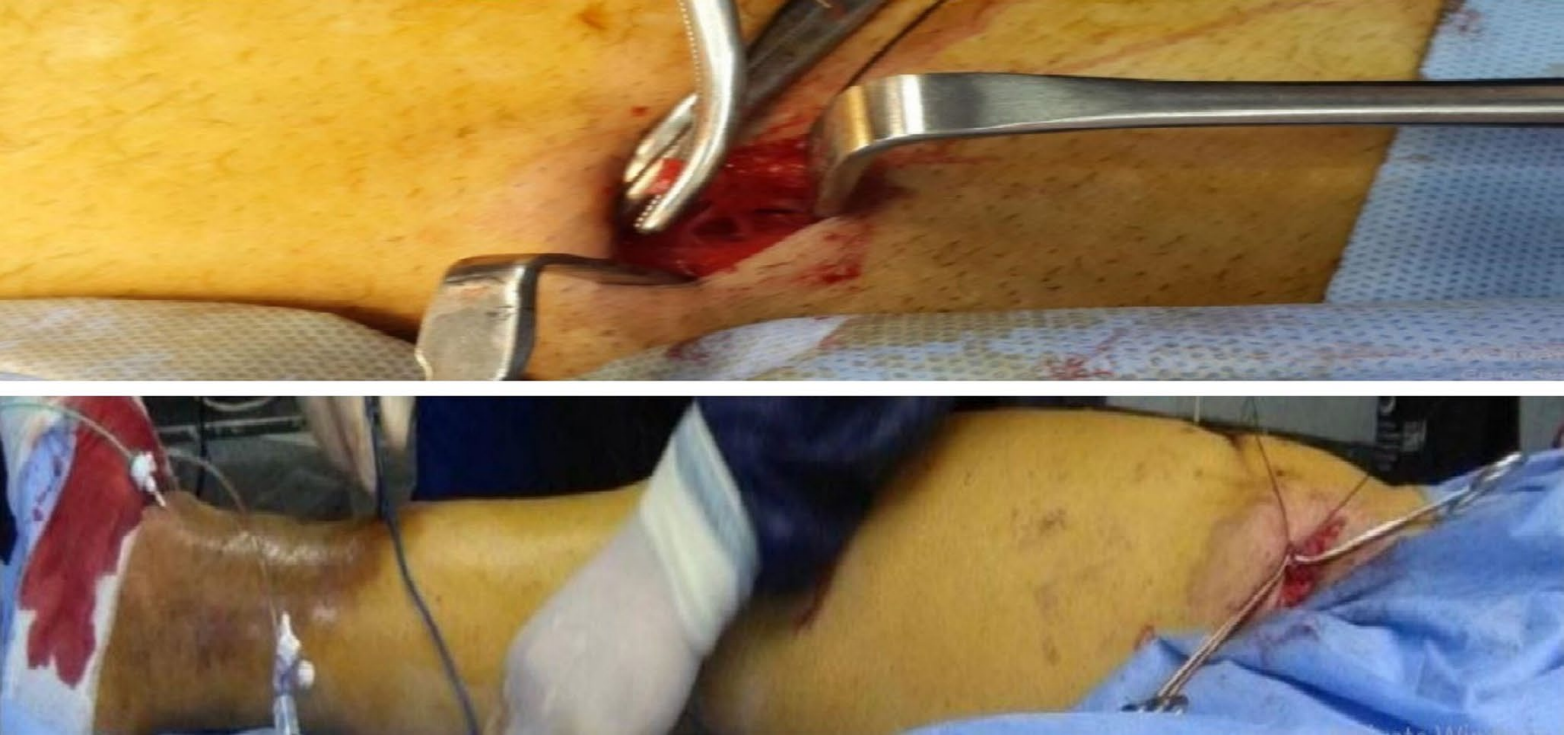
Flush saphenofemoral disconnection (Trendelenburg’s operation) and the introduction of a 0.035 guide wire in the GSV from the medial malleolus, which can be noticed at the groin, then a 5Fr catheter was advanced

**Fig. 2:**
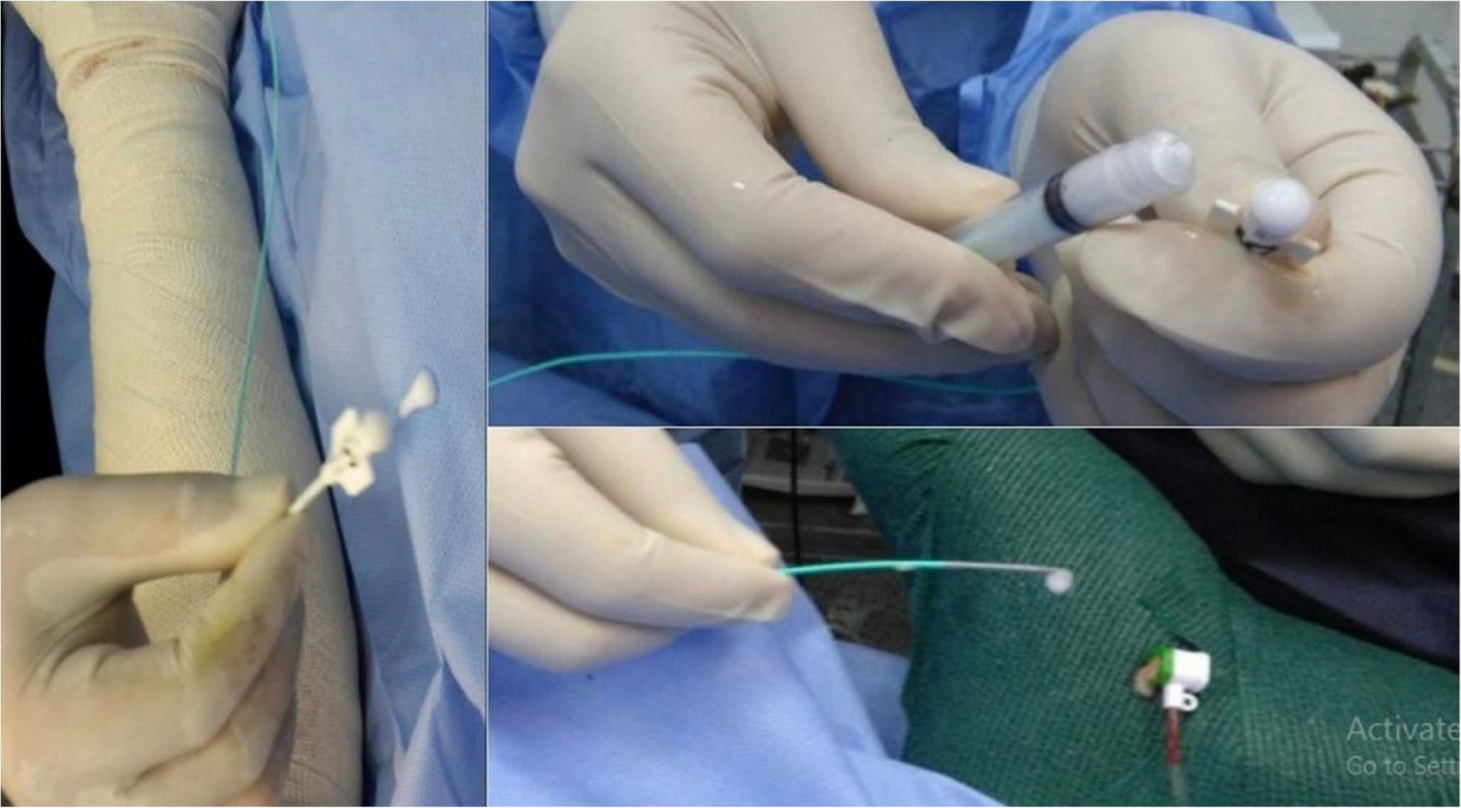
Empty vein foam injection and the spontaneous reversed foam sign

Under local anesthesia, patients were positioned supine, and the targeted area was sterilized. Trendelenburg’s operation was performed, involving flush disconnection of the SFJ and ligation of all tributaries at the groin. Access to the GSV was obtained via needle insertion above the medial malleolus, followed by the introduction of a guide wire advanced toward the groin. The needle was removed, and the catheter was then threaded over the wire, which was subsequently removed. The catheter was retracted 2 cm from the saphenous vein’s termination, and the GSV was closed using Vicryl transfixation and ligation. The catheter and sheath were flushed with saline solution, and the groin wound was closed, dressed, and gauzed along the GSV course (external compression) (Fig. 1). Compression bandages were applied from foot to groin using the empty vein technique during foam injection. A polidocanol 3% foam was prepared in a 1:4 ratio with air, combining 0.5 mL of polidocanol with 2 mL of air in a 3 mL syringe. The foam was injected at a rate of 2.5 mL/ 10 cm of GSV length, with the catheter withdrawn at a speed of 10 cm/min to ensure even foam distribution along the vein endothelium, where this volume and rate were applied on a trial basis (Fig. 2). This process was repeated until the catheter and sheath were fully removed. Technical success was confirmed by the presence of the spontaneous reversed foam sign (SRFS = 3 S), indicating stable foam, sufficient volume, and confinement within the superficial system/GSV. The maximum foam volume ranged from 15 to 20 mL, depending on the length of GSV, as well as concomitant injection of extra truncal varicosities.

## Outcomes

The primary outcome measured was the success rate of GSV ablation, with secondary outcomes including postoperative complications, pain reduction, and factors affecting the success rate.

### Postoperative Follow-up

**Fig. 3:**
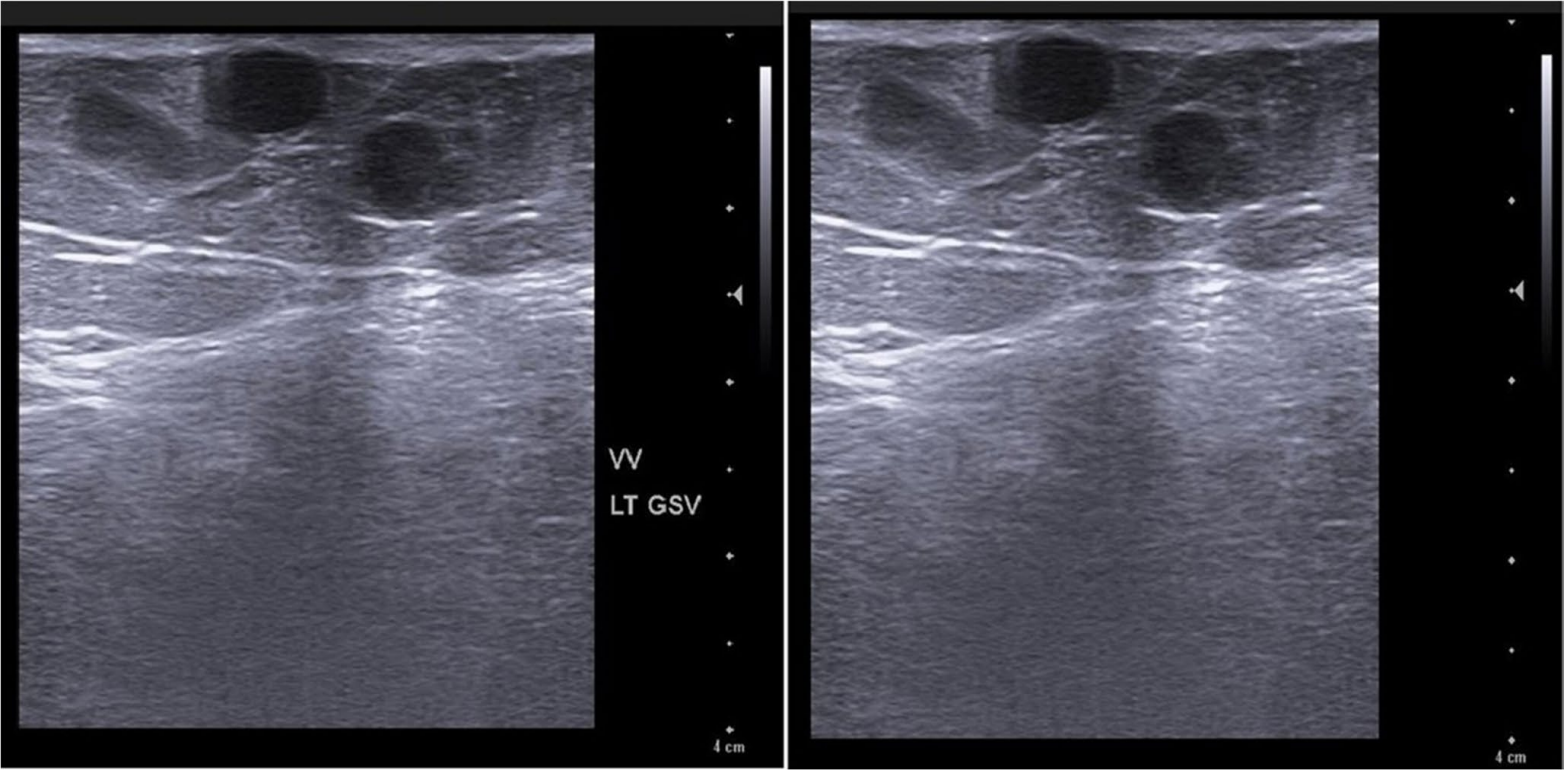
Multiple dilated tortuous superficial varicose veins along the course of the great saphenous vein

**Fig. 4:**
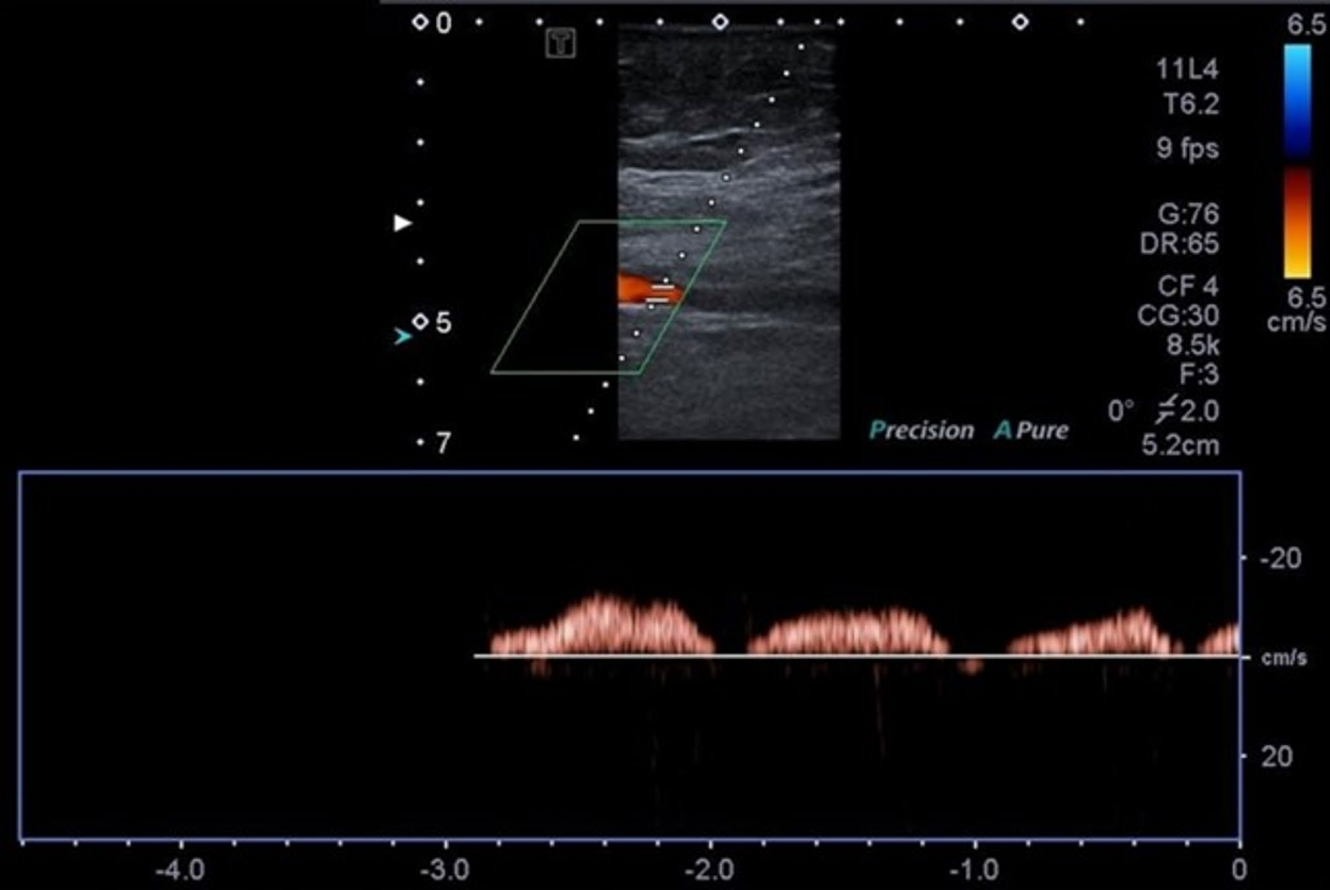
Color Doppler of the LT superficial femoral vein shows patent with normal venous flow pattern, phasic with respiration

Postoperatively, patients rested for six hours before resuming daily activities. Wounds were dressed with crepe bandages for one week, followed by the use of compression stockings for at least one month. Follow-up assessments were conducted at one week, three months, and one year post-procedure, evaluating the surgical site for subcutaneous hematoma, recurrence, ecchymosis, infection, skin ulceration, burn, nerve injury, skin pigmentation, venous ulcer healing, or persistent pain. Duplex ultrasound was utilized to monitor early or late postoperative complications, including GSV diameter or recanalization, if it occurred (Figs. 3 and 4). Patients with residual extra-truncal varicosities were managed with compression sclerotherapy as a planned adjunctive measure; however, no additional or redo procedures were performed on the GSV itself. Pain was measured using a Visual Analogue Scale (VAS).

### Statistical analysis

Qualitative data were analyzed using the Cochrane Q, Chi-square, or Fisher’s Exact test, with mean scores and percentages compared accordingly. The Kolmogorov-Smirnov test assessed the normality of quantitative data distribution. Data were described using median and interquartile ranges (IQR) and compared via the Mann-Whitney U test. Results were considered significant at a two-tailed P-value < 0.05. The statistical analysis was conducted using SPSS version 29 (IBM Corp., Armonk, NY, USA).

## Results

### Demographic and clinical characteristics

The study included 500 participants with a median age of 36 years (IQR: 34–42 years). The cohort comprised 300 males (60%) and 200 females (40%). Occupational characteristics revealed that 340 participants (68%) held standing jobs. In terms of clinical presentation, 220 participants (44%) presented with lower limb varicosities alone, whereas 140 participants (28%) reported varicosities accompanied by venous leg ulcers, and another 140 participants (28%) experienced varicosities with lower limb pain, as shown in Table 1. No patients were lost to follow-up throughout the study visits.

**Table 1:** Demographic and baseline characteristics of the participants

Variables	*n* (%) or median (IQR) *N* = 500
Age (years)	36 (34–42)
Gender	
Male	300 (60%)
Marital state	
Single	200 (40%)
Married	300 (60%)
Job	
Standing job	340 (68%)
Non-standing job	160 (32%)
Complaint	
Lower limb varicosities	220 (44%)
Varicosities and venous leg ulcer	140 (28%)
Varicosities and lower limb pain	140 (28%)

Continuous data are presented as median (IQR), while categorical ones as n (%). *IQR, interquartile range; n, frequency*

### GSV ablation rate

The postoperative outcomes demonstrated high success rates for GSV ablation across follow-up periods. At one week post-procedure, 440 cases (88%) were fully occluded, while 60 cases (12%) were partially occluded. By three months, the number of fully occluded cases decreased to 380 (76%), with partially occluded cases increasing to 120 (24%). At the one-year follow-up, the success rate improved again, with 440 cases (88%) achieving full occlusion and 60 cases (12%) remaining partially occluded. The differences observed were statistically significant (*P* = 0.001), as shown in Table 2.

**Table 2:** Postoperative outcomes, complications and pain assessment over follow-up periods

Variables	Pre-operative	Post-operative			*P*-value ^a^
		1 week	3 months	1 year	
GSV patency (Success rate)					
Occluded		440 (88%)	380 (76%)	440 (88%)	0.001*^a^
Partially occluded		60 (12%)	120 (24%)	60 (12%)	0.001*^a^
Complications					
Oedema		150 (30%)	100 (20%)	0 (0%)	0.001*^a^
Hyperpigmentation		100 (20%)	50 (10%)	0 (0%)	0.001*^a^
Residual varicosities		200 (40%)	0 (0%)	0 (0%)	0.001*^a^
Pain assessment: VAS Score, median (IQR)	4 (1)	2 (1)	1 (0)	1 (0)	0.001*^b^

*GSV *great saphenous vein, *IQR *interquartile range, *VAS *Visual Analogue Scale

* denotes statistical significance

a, Cochrane Q test; b, Friedman test

### Complications

Postoperative complications significantly decreased over time. At one week, oedema was reported in 150 cases (30%), reducing to 100 cases (20%) at three months and resolving completely by the one-year follow-up (*P* = 0.001). Similarly, hyperpigmentation was observed in 100 cases (20%) at one week, decreasing to 50 cases (10%) at three months, and resolving entirely by one year (*P* = 0.001). Residual varicosities were reported in 200 cases (40%) at one week but resolved completely by the three-month follow-up (*P* = 0.001), as shown in Table 2.

### Change in pain

Pain assessment, measured using the Visual Analogue Scale (VAS), showed a significant reduction over the follow-up periods. Pre-operatively, the median VAS score was 4 (IQR: 1). By one week post-procedure, the median score decreased to 2 (IQR: 1), indicating a notable reduction in pain. Further improvements were observed at three months and one year post-operatively, with the median VAS score reduced to 1 (IQR: 0) in both periods. The reduction in pain over time was statistically significant (*P* = 0.001), as shown in Table 2.

### Factors affecting the success rate

Several factors significantly influenced the success rate of surgery at the 1-week postoperative stage and prior to re-intervention for partially occluded cases. By comparing the patients in the occluded group to those in the partially occluded one, age was a significant variable, with the median age of patients in the occluded group being lower at 36 years (IQR: 34–41) compared to 42 years (IQR: 34–43) in the partially occluded group (*P* = 0.004). Gender significantly impacted the success rates, as all partially occluded cases (100%) were male, whereas the occluded group included 54.5% males and 45.5% females (*P* = 0.001). Occupational factors were significant risk factors for the success rate; 100% of the partially occluded cases involved patients with standing jobs, compared to 63.6% in the occluded group (*P* = 0.001). No significant differences were observed in marital status, patient complaints, hospital stay duration, or postoperative complications such as oedema, hyperpigmentation, and residual varicosities, as shown in Table 3.

**Table 3:** Factors affecting the success rate of the surgery at 1-week postoperative (Before re-intervention of partially occluded cases)

Variables	Success Rate		*P*-value
	Occluded (*N* = 440)	Partially occluded (*N* = 60)	
Age (years), median (IQR)	36 (34–41)	42 (34–43)	0.004*^a^
Gender			
Male	240 (54.5%)	60 (100%)	0.001*^b^
Female	200 (45.5%)	0 (0%)	
Marital state			
Single	180 (40.9%)	20 (33.3%)	0.26^c^
Married	260 (59.1%)	40 (66.7%)	
Job			
Standing job	280 (63.6%)	60 (100%)	0.001*^b^
Non-standing job	160 (36.4%)	0 (0%)	
Complaint			
Varicosities	200 (45.5%)	20 (33.3%)	0.2 ^b^
Ulcer	120 (27.3%)	20 (33.3%)	
Pain	120 (27.3%)	20 (33.3%)	
Hospital Stay (hours), median (IQR)	12 (0)	12 (0)	-
Complications			
Oedema	132 (30%)	18 (30%)	1 ^b^
Hyperpigmentation	88 (20%)	12 (20%)	1 ^b^
Residual varicosities	176 (40%)	24 (40%)	1 ^b^

*IQR *interquartile range

* denotes statistical significance

a, Mann Whitney U test; b, Fisher exact test; c, Chi-square test

## Discussion

In this single-arm open-label interventional study, the novel eCAT operation demonstrated high technical success and favorable clinical outcomes in the management of GSV varicosities. All treated veins were successfully ablated, with either full or partial occlusion confirmed by duplex ultrasound at follow-up, alongside significant reductions in pain scores and resolution of complications within one year. These findings highlight the potential of eCAT as an efficacious, safe, and minimally invasive alternative to conventional surgery, addressing the limitations of traditional high ligation and stripping, which are often associated with higher morbidity and prolonged recovery.

Historically, surgery was the standard approach for treating VV. However, the advent of less invasive procedures has transformed treatment paradigms, offering faster recovery and a quicker return to daily activities compared to traditional methods [15, 16]. These advancements align with the growing preference for minimally invasive interventions.

In this study, 60% of participants were male, likely reflecting the impact of prolonged standing and physically demanding occupations. The relatively young median age of 36 years may be attributed to the high prevalence of occupations requiring prolonged standing within this population. Among all participants, 28% either reported pain or presented with ulcerations. This was consistent with findings by Jutley et al., who reported postoperative pain among all participants [17]. However, Kabnick et al. observed that only 4% and 8% of patients in the laser ablation and surgical groups, respectively, experienced pain, although heaviness was a common complaint in 61% and 75% of these groups. Similar to our findings, Kabnick et al.‘s study reported no significant surgical complications, supporting the safety of these procedures. In all cases, access to the GSV was successfully achieved just anterior to the medial malleolus, with or without duplex guidance. Preoperative Duplex ultrasound confirmed GSV patency and suitability for catheterization. Despite the known tortuosity of the vein, the guide wire was advanced smoothly to the groin in all patients included in this study, and no cases required an alternative access route. These findings highlight the feasibility of distal access in carefully selected patients with confirmed vein patency. Although polidocanol foam carries a potential risk of systemic adverse events, particularly in the presence of right-to-left shunts, no cases of serious systemic complications were observed in our cohort. This may be attributed to careful patient selection and the relatively controlled volume and concentration of sclerosant used. Future studies may consider routine preoperative screening for PFO in high-risk patients to further mitigate potential risks. In our study, patients treated with eCAT resumed daily activities within an average of 4.8 ± 1.7 days. This is consistent with the quick recovery times observed in minimally invasive treatments but differs from the longer recovery period associated with surgical ligation and stripping [18]. Interestingly, Porter et al. reported similar recovery times of approximately 3.2 ± 4 days for both conventional surgery and laser ablation, highlighting potential variability due to socioeconomic, occupational, and cultural factors [15]. Notably, patients often perceive surgeries with visible wounds as requiring longer recovery and rehabilitation.

Pain reduction, as measured by the VAS, was another significant finding. The consistent decrease in VAS scores over the follow-up period aligns with results from Shadid et al., reinforcing the effectiveness of minimally invasive techniques in pain management [19]. However, recurrent VV remain a challenge, often attributed to new vessel formation around the SFJ or the persistence of non-competent tributary veins [20–22]. This highlights the importance of thorough vein management to minimize recurrence risk.

We defined recurrent lower limb VV as the anatomical and radiological reappearance of varicosities following initial technical, clinical, and radiological success of the ablation procedure. The variability in definitions of recurrence across comparative studies may introduce bias in favor of one technique over another, undermining the reliability of long-term efficacy comparisons. In our view, true recurrence should not occur if there has been confirmed technical success (complete and durable ablation of the target vein), clinical success (resolution of symptoms), and radiological success (no residual reflux or vein recanalization). The widely accepted concept of neovascularization as a cause of recurrence should be reconsidered in light of potential procedural shortcomings. Specifically, inadequate flush disconnection at the SFJ, or failure to ligate named and unnamed tributaries, may result in residual pathways for venous reflux, which are then misinterpreted as new vessel formation. Therefore, what is often labeled as “neovascularization” may, in fact, represent compensatory mechanisms due to technical insufficiencies during the initial procedure.

At follow-up conducted one week, three months, and one year post-surgery, 88% of patients demonstrated completely closed veins, with 12% showing partial closure. These outcomes were consistent across the study period, emphasizing the durability of eCAT. Factors contributing to recurrence included inefficiency of perforator veins, reopening of the GSV, and reflux in the anterior accessory GSV [22–24].

The observed improvement in GSV occlusion rates over time—from partial to complete occlusion—was confirmed through serial clinical assessments and Duplex ultrasound evaluations at one week, three months, and one year postoperatively. All patients attended scheduled follow-up visits and underwent repeat imaging to ensure accurate assessment of vein closure status. The increase in full occlusion rates by the one-year mark is likely attributable to progressive endothelial fibrosis and delayed closure in cases that were initially partially occluded. Importantly, no adjunctive or redo procedures were performed on the GSV itself. However, a proportion of patients received post-operative compression sclerotherapy specifically targeting extra-truncal residual varicosities. This adjunctive measure, performed during the follow-up period, contributed to the resolution of visible varicosities without requiring additional endovenous interventions. The combination of effective GSV ablation and targeted post-operative sclerotherapy likely underlies the complete clinical resolution observed in these cases.

Endovenous techniques, such as endovenous laser ablation (EVLA), have shown comparable or superior efficacy to traditional surgery regarding complications, pain management, and recovery time, as reported by McBride Rautio et al. [24]. Saphenous vein ablations are emerging as viable replacements for vein stripping and phlebectomy, although their efficacy is enhanced when combined with Trendelenburg’s operation to address the risk of recurrence, particularly in younger patients [25, 26].

Compared to conventional techniques, the eCAT procedure offers several distinct clinical and procedural advantages. Notably, eCAT enables ablation along the entire length of the GSV, including the distal segments in the leg, which may enhance healing outcomes for venous leg ulcers by ensuring complete reflux control [27, 28]. The technique also allows for the management of associated conditions such as saphena varix and labial varicosities, particularly when these arise from SFJ incompetence. Unlike thermal ablation techniques, eCAT does not require tumescent anesthesia or thermal energy, thereby eliminating the risk of saphenous nerve injury and thermal skin burns. This significantly enhances the safety profile of the procedure, especially in treating below-knee varicosities. Moreover, eCAT facilitates the simultaneous treatment of extra-truncal tributaries via polidocanol foam sclerotherapy, streamlining patient care and potentially reducing recurrence.

Clinical outcomes further reinforce these procedural benefits. Patients undergoing eCAT typically experience less pain and ecchymosis compared to traditional surgery and thermal ablation, and they exhibit no postoperative hematoma formation. Recovery is expedited, with most patients discharged on the same day, aligning with the early recovery profiles seen with endovenous techniques. Additionally, eCAT shows no limitations based on GSV diameter, in contrast to thermal ablation, which may be less effective in larger veins. Importantly, eCAT avoids cosmetic disfigurement and offers a cost-effective solution, which is particularly advantageous in resource-limited settings. Collectively, these features make eCAT a comprehensive, safe, and patient-centered alternative to traditional endovenous thermal ablation techniques and surgical stripping.

### Limitations

This study has several limitations. As a single-arm clinical trial, the lack of a control group limits the ability to directly compare the outcomes of eCAT with other treatment modalities, such as endovenous laser ablation or radiofrequency ablation. Additionally, the seven-year study period may have introduced variability in operator technique or technology, potentially affecting consistency. Although the sample size was relatively large, the study population was restricted to a single region, limiting generalizability to diverse populations. Finally, follow-up was limited to one year, which may not adequately capture long-term recurrence or complications. Future multicenter randomized controlled trials with standardized protocols and extended follow-up are warranted to validate these findings and establish broader clinical applicability.

## Conclusion

The eCAT procedure demonstrates a high success rate in occluding the GSV, with significant reductions in pain and minimal complications. Its minimally invasive nature allows for fast recovery. While these results are promising, further studies with robust designs and extended follow-up periods are essential to establish eCAT as a standard treatment option for VV.

## Data Availability

The datasets generated and/or analyzed during the current study are not publicly available due to institutional data protection policies, but are available from the corresponding author on reasonable request.

## Declarations

The datasets generated and/or analyzed during the current study are not publicly available due to institutional data protection policies, but are available from the corresponding author on reasonable request.

### Patient involvement

No patient involvement in the study trial design or dissemination of results.
